# Effect of socioeconomic status on psychosocial problems in 5- to 6-year-old preterm- and term-born children: the ABCD study

**DOI:** 10.1007/s00787-015-0791-4

**Published:** 2015-11-12

**Authors:** Sanne A. A. de Laat, Marie-Louise Essink-Bot, Aleid G. van Wassenaer-Leemhuis, Tanja G. Vrijkotte

**Affiliations:** Department of Public Health, Academic Medical Center (AMC), Amsterdam, The Netherlands; Youth Health Care, GGD Hart voor Brabant, ‘s-Hertogenbosch, The Netherlands; Department of Neonatology, Academic Medical Center (AMC), Amsterdam, The Netherlands

**Keywords:** Psychosocial problems, Preterm birth, Socioeconomic status, Preschool age

## Abstract

This study aimed at analysing the association between socioeconomic status (SES) and psychosocial problems in preterm- and term-born children. Scores of mothers and teachers on the Strengths and Difficulties Questionnaire (SDQ) regarding 217 preterm-born children (<37 weeks’ gestation, mean 34 weeks) were compared with 4336 term-born children in the Amsterdam Born Children and their Development (ABCD) cohort at age 5–6 years. Associations between SDQ scores and SES (maternal education and perceived income adequacy) were examined with multivariate linear regression analysis. The mean mother-reported total difficulties score was significantly higher for preterm children (6.1 ± 4.7) than for term children (5.2 ± 4.1). After covariate adjustment, this difference was 0.5 (95 % CI 0.0–1.0). For preterm children 16.1 % of the mothers reported psychosocial problems compared with 10.1 % for term children. Lower maternal education and lower income adequacy were significantly related to higher SDQ scores of mothers and teachers. Differences in mothers’ SDQ score between preterm and term children were larger in the high-education (Δ0.9, 95 % CI 0.2–1.5) and high-income group (Δ0.9, 95 % CI 0.3–1.6). No significant differences were found between preterm and term children in the SDQ scores reported by teachers. Low level of maternal education and inadequate income showed a much stronger association with psychosocial problems than preterm birth. No combined effect of low SES and preterm birth was found. This study corroborates the evidence for the strength of the disadvantageous effects of low SES on early psychosocial development.

**Electronic supplementary material:**

The online version of this article (doi:10.1007/s00787-015-0791-4) contains supplementary material, which is available to authorized users.

## Introduction

Preterm birth (gestational age <37 weeks) occurs in 5–10 % of births in Europe [1] and in 7.7 % of births in the Netherlands [2]. Preterm birth is associated with increased mortality and psychiatric morbidity [3]. Very preterm and moderately preterm-born children show more behaviour problems than term born children, even after controlling for perinatal and social risk factors and cognitive performance [4]. More hyperactivity, attention problems and emotional problems are reported in very preterm and moderately preterm children [5–7]. Whereas in very preterm-born children psychosocial problems are known to occur at higher rates than in term born children, this is less well established for moderately preterm-born children.

In the Netherlands only very preterm-born children (gestational age <32 + 0 weeks) are entered in a Neonatal Intensive Care Unit (NICU)-based follow-up program until they are 5 years old. In case of less severe prematurity (gestational age between 32 + 0 and 36 + 6 weeks), children without special medical issues are followed by a paediatrician for only a year or less. Most of the preterm children also get their regular check-ups at the Youth Health Care (YHC). In the Netherlands, the YHC follows and protects the health and development of nearly all children. YHC offers publicly funded preventive programmes (screening, vaccinations, health education and promotion) for all children in the Netherlands from birth to 19 years. For professionals in the YHC it is important to be aware of risk factors for developing psychosocial problems.

Preterm birth is known to be associated with educational disparities [8] and deprivation [9]. In turn, socioeconomic disadvantage (low income, low parent education, unemployment and sole parenthood) is an established risk factor for child psychosocial problems [10]. Inequalities in mental health according to family level of education have been reported [11]. There is evidence that low socioeconomic status (SES), as measured by income, education level and occupational status, may have an adverse influence on child development by exacerbating family stress that reduces the effective functioning of parents. High SES may also promote successful child development through the many investments that higher SES parents are able to make in their children’s well-being [12]. High parental education is linked to more behavioural influences on child development, such as lifestyle choices or parenting styles, and knowledge and skills, whereas parental income relates to economic or material resources (such as food and housing) that families are able to purchase [10]. Few studies have analysed the associations between maternal education and family income adequacy separately regarding psychosocial problems in early school-age children.

Low maternal education is also known to be an important predictor of psychosocial problems [4, 13], poor cognitive outcome [13, 14] and later school readiness [15] in very preterm children. Potijk et al. investigated the effect of a composite SES score, based on education, income and occupation, on the association between moderately preterm birth, and behavioural and emotional problems, at age 4 years and showed significantly higher Child Behaviour Checklist (CBCL) total problem scores among moderately preterm children with low SES than among those with high SES (11.3 vs. 5.1 %) [16]. In the present study we compared the total difficulties scores and subscale scores of mothers and teachers on the Strengths and Difficulties Questionnaire (SDQ) of 5- to 6-year-old children and compared the effect of maternal education and perceived income adequacy separately in preterm- and term-born children. We used data from the Amsterdam Born Children and their Development (ABCD) study, a large prospective population-based cohort. We hypothesized a larger effect of preterm birth on child psychosocial problems in families with a low education or low income level, than in families with a higher education or higher income level.

## Patients and methods

### Study population

This study is part of the ABCD study, an ongoing, multi-ethnic, population-based, prospective birth cohort to examine and determine factors in prenatal and early life that might explain the later health of the child and differences in health between children (http://www.abcd-study.nl). The cohort study design has been described previously [17].

**Fig. 1:**
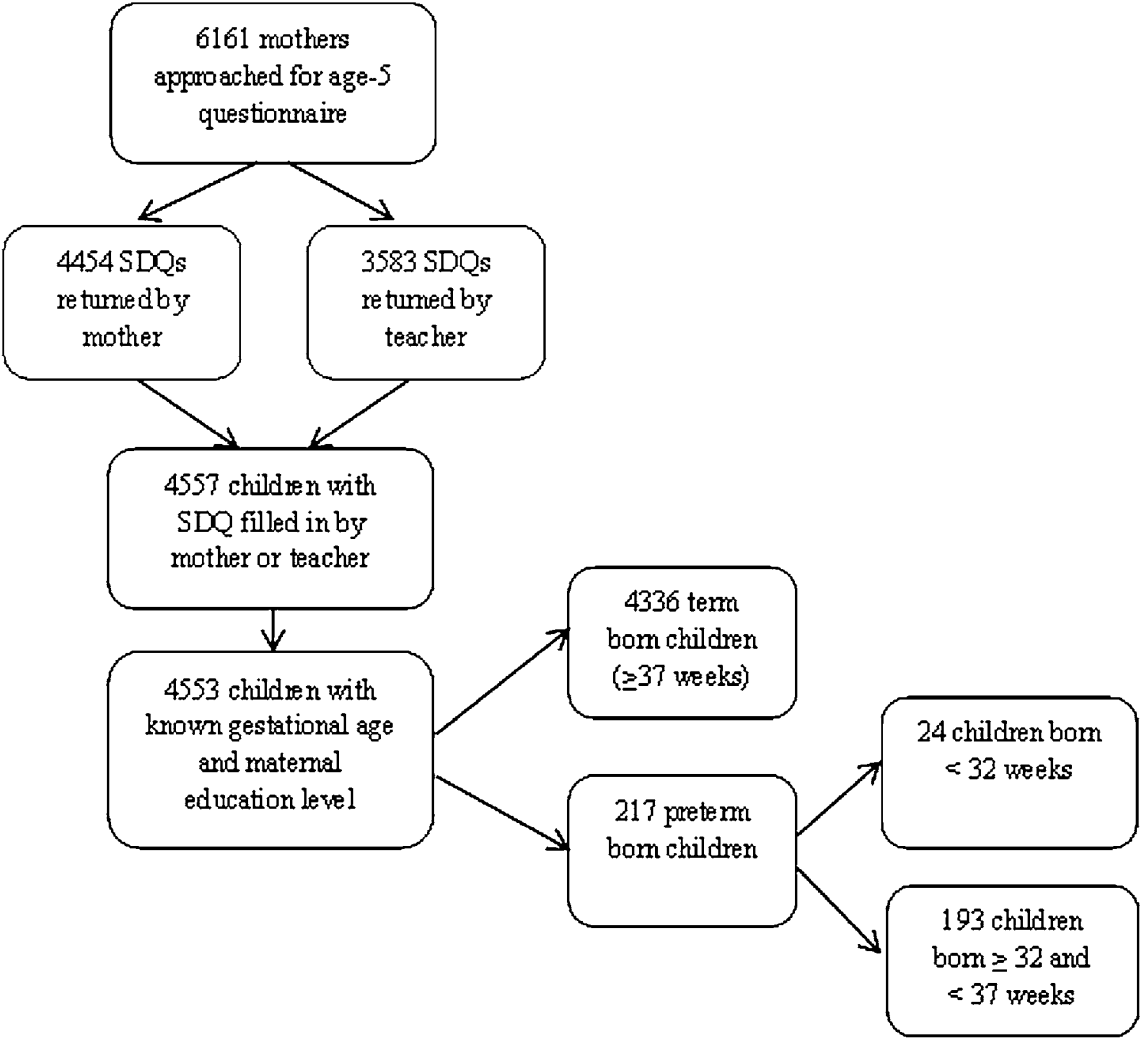
Flowchart of the study population

Figure 1 shows the study procedure and inclusion in the current analyses. For this study we included only those children for whom gestational age, maternal educational level and SDQ data from the mother or the teacher were available. The SDQ was part of a larger 5-year questionnaire filled in by the mother. Twins were excluded from the ABCD study at an earlier stage. Of the 12,373 women approached, 8266 women filled out the pregnancy questionnaire (response rate: 67 %). 6161 mothers who gave permission for follow-up were approached for the 5-year follow-up measurement of their child. For the present study, the final sample consisted of 4553 children (response rate 74 %); mean age 5.2 years, 50.2 % boys and 48.8 % girls. Of these participating children, 4336 were term born (mean gestational age 39 + 5 weeks) and 217 were preterm born (mean gestational age 34 + 3 weeks). Of the 217 preterm-born children, 24 were born very preterm (<32 weeks’ gestation, mean 29 + 1 weeks) and 193 were born moderately preterm (≥32 and <37 weeks’ gestation, mean 35 + 0 weeks).

### SDQ

Psychosocial problems were measured with the Dutch version of the SDQ [18]. The SDQ 25 items divided into five subscales: emotional symptoms, conduct problems, hyperactivity/inattention problems, peer problems and prosocial behaviour. Each item has three response options (not true = 0, somewhat true = 1 and certainly true = 2).The summed score of the first four subscales provides a total difficulties score. A higher total difficulties score indicates a higher risk for psychosocial problems [19]. The Dutch cut-off score for the total difficulties score for the parent-rated SDQ for 7- to 12-year-old children is ≥11; this cut-off score is commonly used in practice in Youth Health Care in the Netherlands [20]. A score of ≥11 means that possible psychosocial problems need further follow-up. SDQ scores can also be used as continuous variables [21]. In the ABCD study the SDQ data were handled following standard procedures; this means that a subscale score was calculated for all children with less than two items missing on that subscale. Further information on the SDQ and scoring is available at http://www.sdqinfo.com. The validity and reliability of the total difficulties score of the parent and teacher SDQ are satisfactory for detecting psychosocial problems in children in a multi-ethnic society [22]. The subscales of the SDQ should be interpreted with caution because the reliability of the SDQ subscales is poorer compared to the total difficulties score, especially in children with a non-Western ethnic background and in 5- to 6-year-old children [21, 22]. In our sample the mother-reported total difficulties score of the SDQ had a Cronbach’s alpha of 0.69, the subscales had alphas of 0.55 for emotional symptoms, 0.46 for conduct problems, 0.75 for hyperactivity/inattention problems, 0.54 for peer problems and 0.63 for prosocial behaviour. These alphas were in line with those found in previous studies [21, 23].

### Socioeconomic status

The self-reported maternal education level and perceived income adequacy were used as separate SES indicators [10]. These data were collected from the 5-year questionnaire. In 42 children, missing information on maternal education level in the 5-year questionnaire was supplemented from the pregnancy questionnaire.

Maternal education level was classified as: high (degree higher vocational education/university), mid (degree higher vocational secondary education/academic secondary education/intermediate vocational education) or low (primary school/technical secondary education/lower vocational secondary education) [24]. Perceived income adequacy was classified as inadequate, adequate or more than adequate income to live.

### Confounding variables

Variables known to be associated with psychosocial problems were analysed as possible confounders. Potential confounders were: gender, ethnic origin, maternal age, parity, smoking during pregnancy, family structure and maternal stress and anxiety. From the pregnancy questionnaire we obtained information on parity (nullipara or multipara), smoking during pregnancy (yes or no) and ethnic origin (Dutch, Turkish, Moroccan, African or other). Ethnic origin was defined by the country of birth of the mother of the pregnant woman [25]. From YHC registration we obtained gender, birth weight and gestational age; small for gestational age was scored based on gender and parity-specific reference curves [26]. From the 5-year questionnaire we obtained age of the mother, family structure (living with both parents or one parent, number of siblings) and maternal anxiety and stress; these were assessed by the Depressive Anxiety and Stress Scale 21 (DASS21) [27].

### Statistical analysis

First, we assessed pregnancy, child and family characteristics categorised by term- and preterm-born children. Differences between preterm- and term-born children were examined using a Chi square test for categorical variables and a *t* test for continuous variables. Characteristics of responders of the 5-year questionnaire were compared with that of non-responders.

Next, we calculated mean and standard deviations (SD) for SDQ total difficulties scores as well as subscale scores across levels of maternal education and perceived income adequacy for term and preterm children. We analysed total difficulties scores and subscales as continuous data, but also calculated % of children scoring above cut-off of the mother-reported SDQ. SDQ differences (total difficulties scores, as well as the subscales) between preterm- and term-born children were assessed with linear regression. SDQ differences between maternal education levels and income levels were assessed with one-way ANOVA (total difficulties scores, as well as the subscales). We performed linear regression models to examine the association between preterm birth and total difficulties score for the total group, followed by stratification by SES level. Linear regression models were only performed on the total difficulties scores, because of concerns regarding the reliability of the subscales of the SDQ in 5- to 6-year-old children [22]. Adjustments were made for gender, number of siblings, one-parent household, Dutch or non-Dutch ethnic origin, age of the mother, smoking during pregnancy, small for gestational age and the DASS21 total score (model 1). In model 2 we additionally adjusted for the other SES indicator (income adequacy or maternal education level). Finally, we assessed whether preterm birth and low SES had multiplicative effects on SDQ scores by adding the preterm birth * SES interaction term. Analyses were performed for SDQ reported by mother and teacher independently. Statistical analyses were performed in SPSS version 21, statistical significance was set at *p* < 0.05 level.

## Results

### Characteristics

Table 1 shows the pregnancy, child and family characteristics of the children in this study. The preterm group included significantly more children of non-Dutch ethnic origin (*p* = 0.023) and low maternal education (*p* = 0.016). The preterm children were more often first born and had less siblings at 5 years of age, than term-born children.

**Table 1:** Characteristics of the study sample according to birth group

Characteristics		Total	Term (37–42 weeks)	Preterm (<37 weeks)	Very preterm (<32 weeks)	Moderately preterm (32–36 weeks)
*N* (%)		4553 (100 %)	4336 (95.2 %)	217 (4.8 %)	24 (0.5 %)	193 (4.2 %)
Pregnancy						
Gestational age	Mean in weeks (SD)	39.46 (1.732)	39.71 (1.247)	34.41 (2.245)	29.13 (1.624)	35.06 (1.197)
Parity	% nullipara	2570 (56.4 %)	2419 (55.8 %)	151 (69.6 %)	16 (66.7 %)	135 (69.9 %)
Any maternal cigarette smoking	Yes	399 (8.8 %)	373 (8.6 %)	26 (12.0 %)	5 (20.8 %)	21 (10.9 %)
Any maternal alcohol use	Yes	1158 (25.4 %)	1124 (26.0 %)	34 (15.7 %)	2 (8.3 %)	32 (16.6 %)
Child						
Gender	Boy	2287 (50.2 %)	2171 (50.1 %)	116 (53.5 %)	13 (54.2 %)	103 (53.4 %)
	Girl	2266 (49.8 %)	2165 (49.9 %)	101 (46.5 %)	11 (45.8 %)	90 (46.4 %)
Birth weight	Mean in gram (SD)	3468.9 (541.5)	3519.8 (482.5)	2451.4 (643.4)	1248.4 (318.8)	2594.8 (507.8)
Small for gestational age (<p10)	Yes	409 (9.0 %)	396 (9.1 %)	13 (6.0 %)	1 (4.2 %)	12 (6.2 %)
Age at current assessment	Mean (SD)	5.2 (0.33)	5.2 (0.33)	5.2 (0.37)	5.4 (0.47)	5.2 (0.36)
Family (at current assessment)						
Maternal education	Low	697 (15.3 %)	649 (15.0 %)	48 (22.1 %)	10 (41.7 %)	38 (19.7 %)
	Mid	990 (21.7 %)	945 (21.8 %)	45 (20.7 %)	5 (20.8 %)	40 (20.7 %)
	High	2866 (62.9 %)	2742 (63.2 %)	124 (57.1 %)	9 (37.5 %)	115 (59.6 %)
Perceived income adequacy	Inadequate	570 (12.5 %)	539 (12.4 %)	31 (14.3 %)	2 (8.4 %)	29 (15.0 %)
	Adequate	1089 (23.9 %)	1036 (23.9 %)	53 (24.4 %)	5 (20.8 %)	48 (24.9 %)
	More than adequate	2716 (59.7 %)	2597 (59.9 %)	119 (54.8 %)	13 (54.2 %)	106 (54.9 %)
	Missing	178 (3.9 %)	164 (3.8 %)	14 (6.5 %)	4 (16.7 %)	10 (5.2 %)
Ethnic origin	Dutch	2968 (65.2 %)	2842 (65.5 %)	126 (58.1 %)	11 (45.8 %)	115 (59.6 %)
	Turkish	156 (3.4 %)	147 (3.4 %)	9 (4.1 %)	1 (4.2 %)	8 (4.1 %)
	Moroccan	271 (6.0 %)	264 (6.1 %)	7 (3.2 %)	2 (8.3 %)	5 (2.6 %)
	African	234 (5.1 %)	214 (4.9 %)	20 (9.2 %)	2 (8.3 %)	18 (9.3 %)
	Other: Western	549 (12.1 %)	518 (12.0 %)	31 (14.3 %)	3 (12.5 %)	28 (14.5 %)
	Other: Non-Western	373 (8.2 %)	349 (8.1 %)	24 (11.1 %)	5 (20.8 %)	19 (9.8 %)
Maternal age	Mean in years (SD)	37.4 (5.4)	37.4 (5.3)	36.9 (6.5)	34.6 (9.2)	37.2 (6.1)
DASS21 score	Mean (SD)	4.5 (5.6)	4.4 (5.5)	5.1 (7.6)	4.9 (6.2)	5.2 (7.7)
Siblings	No	749 (16.5 %)	696 (16.1 %)	53 (24.4 %)	7 (29.2 %)	46 (23.8 %)
	1	3169 (69.6 %)	3032 (69.9 %)	137 (63.1 %)	13 (54.2 %)	124 (64.2 %)
	2	475 (10.4 %)	455 (10.5 %)	20 (9.2 %)	2 (8.3 %)	18 (9.3 %)
	3 or more	54 (1.2 %)	54 (1.2 %)	0	0	0
Living with	One parent	427 (9.4 %)	403 (9.3 %)	24 (11.1 %)	4 (16.7 %)	20 (10.4 %)

Responders (*n* = 4553) to the 5-year questionnaire were compared with non-responders (*n* = 1604), as shown in an additional table in Online Resource 1. Non-responders were more often of non-Dutch ethnicity (*p* < 0.001), had less years of maternal education (*p* < 0.001), had a shorter gestational age in weeks (*p* = 0.005) and their children had a lower birth weight (*p* < 0.001). Prematurity was not more common in the non-responder group (*p* = 0.293).

### Preterm birth and psychosocial problems

Table 2 shows the unadjusted mean SDQ scores for term- and preterm-born children. Overall, the mean total difficulties score reported by mothers was 5.3 ± 4.1. Mothers reported a higher mean score on the total difficulties scale for preterm children compared to term children (*p* = 0.003). Mothers reported higher SDQ subscale scores in preterm children on emotional symptoms (*p* = 0.004), hyperactivity/inattention (*p* = 0.011) and peer problems (*p* = 0.009). Teacher SDQ scores followed almost the same trends, but differences between preterm children and term children were non-significant. For term-born children 10.1 % of the mothers reported scores that were above the Dutch cut-off (≥11) for the total difficulties score; for preterm-born children 16.1 % of the mothers reported scores above cut-off (*p* = 0.004). The difference in SDQ total difficulties score between very preterm children (<32 weeks’ gestation) and term children (Δ1.8, 95 % CI 0.1–3.5) was larger than the difference between moderately preterm children (≥32 and <37 weeks’ gestation) and term children (Δ 0.7, 95 % CI 0.1–1.3).

**Table 2:** Mean Strengths and Difficulties Questionnaire (SDQ) scores in preterm- and term-born children reported by mothers and teachers divided by maternal education level

Maternal education level		Term				Preterm (<37 weeks)				*p*-value birth group^a^	*p*-value educa-tion^b^
		All (*N* = 4240)	Low (*N* = 617)	Mid (*N* = 902)	High (*N* = 2721)	All (*N* = 211)	Low (*N* = 46)	Mid (*N* = 41)	High (*N* = 124)		
SDQ score mother	% SDQ total difficulties score ≥11	10.1 %	21.0 %	13.0 %	6.5 %	16.1 %	25.0 %	15.6 %	12.9 %	0.004*	<0.001*
	Total difficulties	5.2 (4.1)	7.6 (4.6)	6.0 (4.3)	4.4 (3.6)	6.1 (4.7)	7.7 (4.9)	6.7 (5.1)	5.3 (4.3)	0.003*	<0.001*
Subscales	Emotional symptoms	1.0 (1.3)	1.2 (1.4)	1.0 (1.4)	0.9 (1.2)	1.2 (1.4)	1.3 (1.5)	1.2 (1.4)	1.2 (1.3)	0.004*	<0.001*
	Conduct problems	1.1 (1.3)	1.5 (1.5)	1.2 (1.3)	0.9 (1.2)	1.0 (1.3)	1.2 (1.4)	1.0 (1.2)	1.0 (1.3)	0.743	<0.001*
	Hyperactivity/inattention	2.4 (2.1)	3.1 (2.2)	2.9 (2.3)	2.1 (2.0)	2.8 (2.5)	3.4 (2.5)	3.5 (2.6)	2.3 (2.4)	0.011*	<0.001*
	Peer Problems	0.8 (1.3)	1.7 (1.6)	1.0 (1.4)	0.6 (1.1)	1.1 (1.4)	1.7 (1.6)	1.0 (1.3)	0.8 (1.3)	0.009*	<0.001*
	Prosocial	8.0 (1.8)	7.8 (1.9)	8.0 (1.7)	8.1 (1.7)	7.9 (1.8)	7.4 (1.9)	8.5 (1.6)	7.8 (1.7)	0.260	<0.001*

Maternal education level		Term				Preterm (<37 weeks)				*p*-value birth group^a^	*p*-value educa-tion^b^
		All (*N* = 3414)	Low (*N* = 450)	Mid (*N* = 208)	High (*N* = 2226)	All (*N* = 166)	Low (*N* = 29)	Mild (*N* = 35)	High (*N* = 102)		
SDQ score teacher	Total difficulties	5.3 (4.7)	7.0 (5.6)	5.5 (4.9)	4.8 (4.3)	5.9 (5.0)	8.2 (6.0)	6.2 (5.6)	5.1 (4.2)	0.101	<0.001*
Subscales	Emotional symptoms	1.2 (1.6)	1.4 (1.7)	1.1 (1.6)	1.1 (1.6)	1.4 (1.7)	2.0 (2.4)	1.2 (1.6)	1.2 (1.6)	0.142	0.001*
	Conduct problems	0.8 (1.3)	1.2 (1.6)	0.9 (1.3)	0.7 (1.2)	0.8 (1.3)	1.0 (1.8)	1.0 (1.5)	0.7 (1.0)	0.938	<0.001*
	Hyperactivity/inattention	2.3 (2.6)	3.0 (2.9)	2.6 (2.8)	2.1 (2.4)	2.6 (2.7)	3.7 (2.5)	3.3 (3.3)	2.1 (2.3)	0.131	<0.001*
	Peer problems	1.0 (1.4)	1.4 (1.7)	0.9 (1.4)	0.9 (1.3)	1.1 (1.4)	1.5 (1.6)	0.8 (1.3)	1.1 (1.3)	0.275	<0.001*
	Prosocial	7.6 (2.2)	7.3 (2.3)	7.6 (2.2)	7.7 (2.1)	7.5 (2.2)	7.2 (2.2)	7.5 (2.2)	7.6 (2.3)	0.425	<0.001*

Mean (SD), * = significant

^a^Tested for differences in mean scores between all term and preterm children using linear regression

^b^Tested for differences in mean scores between children with low, mid and high maternal education level using one-way ANOVA

### SES, preterm birth and psychosocial problems

**Fig. 2:**
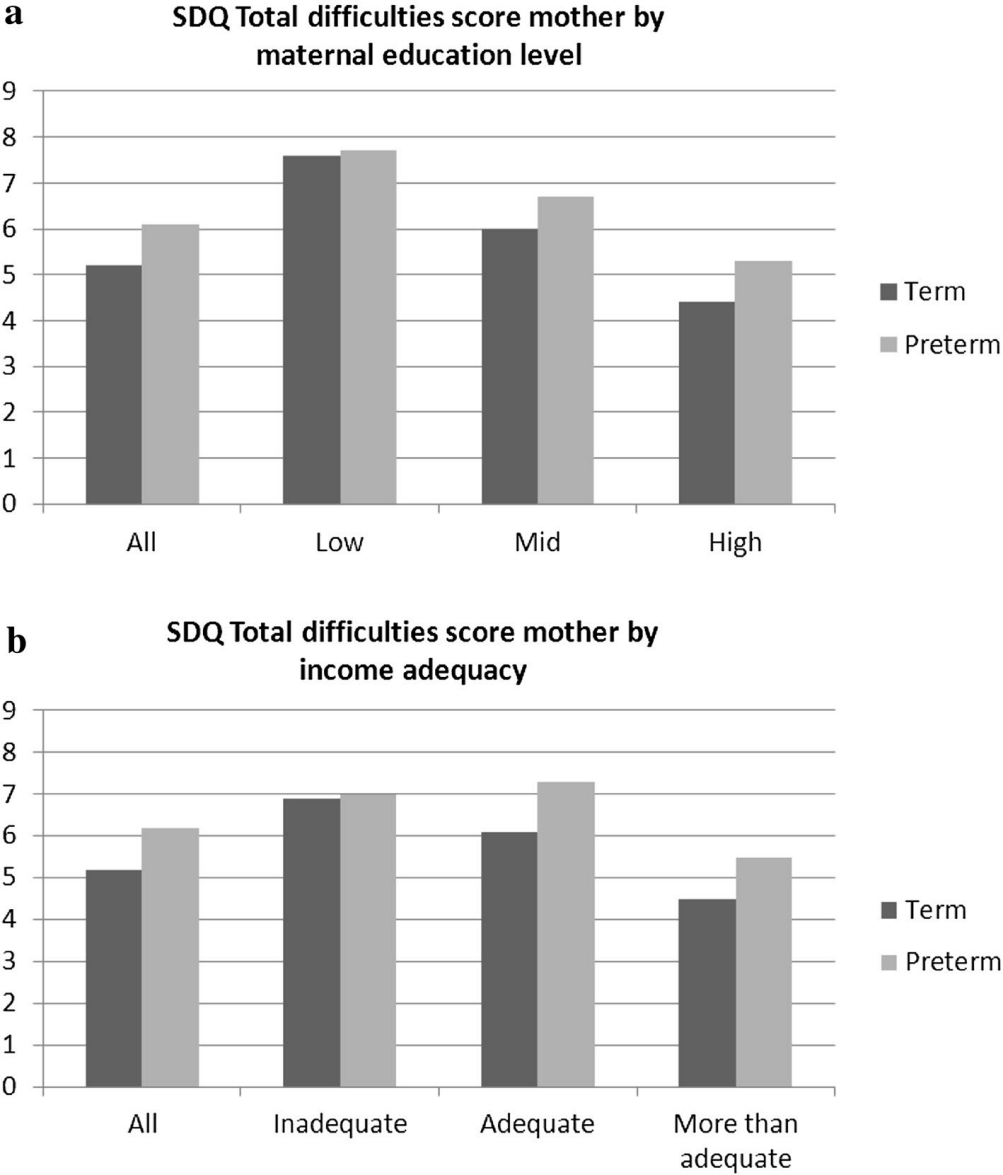
Mean mother-reported SDQ total difficulties score by maternal education level (**a**) and perceived income adequacy (**b**)

All differences in mean SDQ scores of mothers and teachers according to level of maternal education were significant (*p* ≤ 0.001). Mothers with a low education level or perceived income inadequacy reported more psychosocial problems in both the term and preterm group (Fig. 2). The highest SDQ scores were reported in preterm children with a low maternal education level or inadequate income. Table 3 shows the differences between term and preterm children on SDQ total difficulties score after the addition of the covariates in the two models. After adjustment for gender, siblings, one-parent household, ethnic origin, age of the mother, smoking during pregnancy, small for gestational age and DASS21 score (model 1), the difference between the mean SDQ total difficulties score of mothers in preterm children and term children became non-significant (0.5, 95 % CI 0.1–1.0). Differences in SDQ total difficulties score between preterm and term children were largest in mothers with a high education level. After adjustment, only differences between preterm and term children in the highly educated group remained significant (Δ0.9, 95 % CI 0.2–1.5). Differences between preterm and term children from low educated mothers appeared larger when the teacher reported psychosocial problems; however, the difference was not significant. When comparing differences in SDQ total difficulties score of mothers by level of income adequacy, there was a significant difference between preterm and term children in the ‘more than adequate income’ group (Δ1.0, 95 % CI 0.3–1.6); this difference remained significant after adjustment (Δ0.9, 95 % CI 0.3–1.6) (Table 4).

**Table 3:** Differences in Strengths and Difficulties Questionnaire (SDQ) total difficulties score between preterm and term born children divided by maternal education level in two regression models and interaction

	Maternal education level	Term	Preterm	Difference (95 % CI)	Model 1 Adjusted difference (95 % CI)	Model 2 Adjusted difference (95 % CI)	Inter-action *P* ^a^
Total SDQ score mother	All	5.2 ± 4.1	6.1 ± 4.7	0.9 (0.3, 1.4)*	0.5 (−0.1, 1.0)	0.5 (0.0, 1.1)	0.320
	Low	7.6 ± 4.6	7.7 ± 4.9	0.1 (−1.3, 1.2)	−0.1 (−1.5, 1.3)	−0.2 (−1.6, 1.2)	
	Mid	6.0 ± 4.3	6.7 ± 5.1	0.7 (−0.7, 2.1)	0.1 (−1.3, 1.4)	0.1 (−1.3, 1.5)	
	High	4.4 ± 3.6	5.3 ± 4.3	0.9 (0.2, 1.5)*	0.8 (0.2, 1.5)*	0.9 (0.2, 1.5)*	
Total SDQ score teacher	All	5.3 ± 4.7	5.9 ± 5.0	0.6 (−0.1, 1.4)	0.4 (−1.1, 3.2)	0.5 (−0.2, 1.2)	0.497
	Low	7.0 ± 5.6	8.2 ± 6.0	1.2 (−1.0, 3.3)	1.1 (−1.1, 3.2)	1.3 (−0.9, 3.5)	
	Mid	5.5 ± 4.9	6.2 ± 5.6	0.7 (−1.0, 2.4)	0.5 (−1.2,2.3)	0.8 (−1.0, 2.7)	
	High	4.8 ± 4.3	5.1 ± 4.2	0.3 (−0.6, 1.1)	0.1 (−0.7, 1.0)	0.2 (−0.7, 1.0)	

SDQ scores are presented as mean ± SD

*Model 1* corrected for gender, siblings, one-parent household, Dutch ethnic origin, age of mother, smoking during pregnancy, small for gestational age, and DASS21 total score

*Model 2* corrected for model 1 + income adequacy

* = significant (*p* < 0.05)

^a^Interaction between prematurity and maternal education level

**Table 4:** Differences in Strengths and Difficulties Questionnaire (SDQ) total difficulties score and between preterm- and term-born children divided by perceived income adequacy in two regression models and interaction

	Income adequacy	Term	Preterm	Difference (95 % CI)	Model 1 Adjusted difference (95 % CI)	Model 2 Adjusted difference (95 % CI)	Inter-action P^a^
Total SDQ score mother	All	5.2 ± 4.1	6.2 ± 4.7	1.0 (0.4, 1.5)*	0.6 (0.0, 1.1)	0.5 (0.0, 1.1)	0.072
	Inadequate	6.9 ± 5.0	7.0 ± 4.9	0.1 (−1.8, 1.7)	−1.2 (−2.9, 0.5)	−1.4 (−3.0, 0.3)	
	Adequate	6.1 ± 4.4	7.3 ± 5.0	1.2 (0.0, 2.4)	0.6 (−0.6, 1.8)	0.4 (−0.8, 1.6)	
	More than adequate	4.5 ± 3.6	5.5 ± 4.4	1.0 (0.3, 1.6)*	0.9 (0.3, 1.6)*	0.9* (0.3, 1.6)	
Total SDQ score teacher	All	5.2 ± 4.6	5.9 ± 5.0	0.7 (−0.1, 1.5)	0.5 (−0.2, 1.3)	0.5 (−0.2, 1.2)	0.631
	Inadequate	6.2 ± 5.3	7.2 ± 6.7	1.0 (−1.3, 3.3)	0.2 (−2.1, 2.4)	0.2 (−2.1, 2.4)	
	Adequate	5.8 ± 5.0	5.6 ± 4.2	−0.2 (−1.8, 1.4)	0.0 (−1.6, 1.6)	−0.1 (−1.7, 1.5)	
	More than adequate	4.8 ± 4.3	5.8 ± 4.9	1.0 (0.0, 1.8)	0.8 (−0.1, 1.7)	0.8 (−0.1, 1.7)	

SDQ scores are presented as mean ± SD

*Model 1* corrected for gender, siblings, one-parent household, Dutch ethnic origin, age of mother, smoking during pregnancy, small for gestational age and DASS21 total score

*Model 2* corrected for model 1 + maternal education level

* = significant (*P* < 0.05)

^a^Interaction between prematurity and income adequacy

### Interaction between preterm birth and SES on psychosocial problems

The interaction between preterm birth and perceived income adequacy on SDQ total difficulties score reported by mothers was borderline significant (*p* = 0.072). No other preterm birth * SES interactions were found (maternal education SDQ mother *p* = 0.320, maternal education SDQ teacher *p* = 0.497, income adequacy SDQ teacher *p* = 0.631).

## Discussion

This study showed that mothers of 5- to 6-year-old preterm children (mean gestational age of 34 weeks) reported significantly more psychosocial problems than mothers of term-born children. For teacher-rated psychosocial problems we found similar trends. Mothers with a low education level or low perceived income adequacy reported more difficulties in both term- and preterm-born children and their SDQ scores were also most often in the (sub)clinical range (21–25 %), indicating a higher risk for psychosocial problems. Differences in mother-reported SDQ scores between preterm and term children were larger in the highly educated and ‘more than adequate’ income group. In contrast to our hypothesis, no combined effects of preterm birth and SES were found. Maternal education level and perceived income adequacy were strong indicators of psychosocial problems, overruling effects of preterm birth.

The strong association between low SES and child problem behaviour has been found before [10, 11, 21]. Combined effects of SES and preterm birth on behavioural and emotional problems were analysed previously by Potijk et al. [16] and Potharst et al. [13]. In contrast to our study, Potijk et al. showed that low SES and moderately preterm birth had independent, multiplicative negative effects on behavioural and emotional problems in 4-year-old children, especially in girls. Their study combined education, income and occupation in a composite SES score and did not report the effect of the separate SES indicators, thus limiting the understanding of the unique contribution of the SES indicators [12]. Potharst et al. [13] found a trend for higher SDQ parent scores in very preterm children (mean gestational age of 28 weeks) with low parental education. Contrary to our findings the difference in SDQ total difficulties scores between the very preterm and term group was most pronounced in the lowest parental education sub group. We showed that psychosocial problems were more common in low SES children, but (moderately) preterm birth did not multiply this effect.

As expected [9, 28], preterm birth was more common in low SES families. In our study 22 % of the preterm-born children had a mother with a low education level, in comparison with 15 % of the term-born children. Differences in educational background between mothers of preterm- and term-born children could be explained by differences in exposure to risk factors, such as maternal smoking, maternal obesity, teenage pregnancy and psychosocial stress [8, 28]. These risk factors lead also to less optimal circumstances after birth and exposure to multiple stressful experiences early in life may explain the higher rates of psychosocial problems in low SES groups. The perinatal period is a critical period for child development, with lifelong effects on physical and mental wellbeing. There is growing evidence that exposures during pregnancy, such as lifestyle factors and maternal mental health, are predictive of child behavioural, emotional and learning outcomes [29]. The activation of the Hypothalamic Pituitary Adrenal axis by elevated maternal cortisol is suggested to be one of the main biological mechanisms underlying the effects of antenatal depression on offspring adversity [30]. Also antenatal anxiety and parental stress are related to children’s problem behaviour [31, 32].

In this study we controlled for risk factors such as maternal depression and anxiety, smoking during pregnancy and maternal age. Controlling for these confounders rendered the differences in SDQ scores between preterm and term born children non-significant. This suggests that confounders like maternal psychopathology play an important role in the association between preterm birth and psychosocial problems in children, which is also described by others [33].

Our study underlines the important disadvantageous effects of low SES for both preterm- and term-born children. Parents with low SES have fewer resources resulting in less capacity for supportive, consistent and involved parenting. Parents with an higher level of education might have more knowledge, this could result in a different approach towards their children, which may prevent or diminish problem behaviour in children [34]. This possibly explains why we only found more psychosocial problems in preterm children than in term children in the highly educated and ‘more than adequate’ income group. In the children with high SES we more clearly found a negative effect of prematurity on psychosocial problems. Low SES seemed to overrule the effect of preterm birth.

Another explanation could be that highly educated mothers were more aware of behaviour problems of their preterm children or alternatively have lower acceptance. Teachers did not report more psychosocial difficulties for these preterm children. The more the mother is convinced that her very preterm child is vulnerable, the more psychosocial problems she may report. Additionally, the interaction between mothers and (very) preterm children may be more difficult, especially when the child faces developmental disabilities. Potharst et al. describe that mothers with socioeconomic disadvantages, raising a very preterm child with severe disabilities, struggle most with giving adequate sensitive support for the autonomy development of their child at 5 years of age [35]. The relative contribution of each of these three potential explanations of the disadvantageous effects of low SES on early school-age psychosocial development remains to be elucidated.

### Strengths and limitations

A strength of this study is the longitudinal information on (risk factors in) pregnancy, birth and first years of life. Also, we used both parental and teacher reports to assess psychosocial problems. We found an interrater correlation of 0.40, which is comparable to the interrater correlation found by Goodman (*r* = 0.46) [36]. This low to moderate agreement is common and indicates the importance of multiple informants, because children may show different problematic behaviour in the home or the school situation, depending on the situational context and the person they are interacting with. Apart from different behaviour, the informants also have different possibilities to observe specific behaviour in their context [37]. There is no gold standard available for the integration of multiple assessments in the diagnostic process. If information from parents and teachers is available, instead of looking at discrepancy effects, it seems more important to consider additive informant effects as a predictor of outcome [38].

Furthermore, the SES measures we used are frequently used indicators of SES. Because maternal education and income adequacy do not completely overlap, analysing these two SES indicators separately provides additional information [39]. In our study, only 26 % of the mothers with a low education level had perceived inadequate income. We found higher SDQ scores (indicating more psychosocial problems) for children with low maternal education than for children with inadequate income at home. There was a larger difference between total difficulties scores of children with low and highly educated mothers than between children with an inadequate income and more than adequate income. This confirms the hypothesis that maternal education is linked to more behavioural influences on child development than income adequacy [10].

Our study also has some limitations. First, our non-response analysis showed selective response, with a higher participation rate among highly educated mothers. Although this may not have biased our results, our reported socioeconomic inequalities may be an underestimation of the actual effects. Second, the ABCD cohort had a low percentage (4.8 %) of preterm children compared with the preterm birth prevalence in the Dutch population (7.7 %) [2] because twins were excluded from the study at an earlier stage. The preterm group was relatively small (217 preterm children); 21.8 % of the preterm children had mothers with low maternal education and 17.5 % with inadequate income. Our data did not allow to further analyse differences between very preterm and moderately preterm children because of low power (*n* = 24 and *n* = 193). Third, in our study we used maternal education level and perceived income adequacy at age 5–6 years when SDQ was rated. This might be different from the situation during pregnancy for some women. Finally, the SDQ must be seen as a screening tool, rather than a diagnostic instrument for psychosocial problems. Further evaluation of psychosocial problems is necessary. We choose to show corrected models for the total difficulties scores only, because there are concerns regarding the reliability of the subscales of the SDQ in the 5- to 6-year-old children [22].

### Implications for Youth Health Care (YHC)

This study underlines the disadvantageous effects of low SES on psychosocial development. However, we found no evidence to support a change in the existing YHC guideline that describes detection of psychosocial problems in preterm children [40]. Early detection and treatment of psychosocial problems may lead to considerable benefits regarding child development, well-being and health. Further research is needed to explore whether suitable interventions are available for children with low SES to prevent psychosocial problems. Based on the present study, we recommend YHC to be extra alert for psychosocial problems in low SES families, irrespective of term or (moderately) preterm birth.

## Electronic supplementary material

Below is the link to the electronic supplementary material.

Table responder/nonresponder (DOC 46 kb)
