# Supplementary material for: Effect of socioeconomic status on psychosocial problems in 5- to 6-year-old preterm- and term-born children: the ABCD study
Source: Eur Child Adolesc Psychiatry. 2015 Nov 12;25:757–67. doi: 10.1007/s00787-015-0791-4 (PMC4932131; doi:10.1007/s00787-015-0791-4)
Supplement: Supplementary file 1 — Table responder/nonresponder (DOC 46 kb) [file 787_2015_791_MOESM1_ESM.doc]

| **Characteristics** |  | **Responder SDQ mother and/or teacher** | **Non-responder SDQ mother and/or teacher** | **P-value** |
| --- | --- | --- | --- | --- |
|  |  | n=4553 (73.9%) | n=1604 (26.1%) |  |
| **Pregnancy** | |  |  |  |
| Gestational age | Mean in weeks (SD)  Premature (<37 weeks) Early premature (<32 weeks)  Mild premature (32-37 weeks) | 39.46 (1.732)  217 (4.8%)  24 (0.5%)  193 (4.2%) | 39.32 (1.824)  87 (5.4%)  12 (0.7%)  75 (4.7%) | 0.005^*^  0.293  0.317  0.458 |
| Parity | % nullipara | 56.4% | 53.3% | 0.029^*^ |
| Maternal cigarette smoking | Yes | 399 (8.8%) | 200 (12.5%) | <0.001^*^ |
| Maternal alcohol use | Yes | 1158 (25.4%) | 248 (15.4%) | <0.001^*^ |
| **Child** | |  |  |  |
| Gender | Boy | 2287 (50.2%) | 783 (48.8%) | 0.330 |
|  | Girl | 2266 (49.8%) | 821 (51.2%) |  |
| Birth weight | Mean in gram (SD) | 3468.9 (541.5) | 3406.9 (569.1) | <0.001^*^ |
| Small for gestational age (<p10) | Yes | 409 (9.0%) | 158 (9.8%) | 0.380 |
| Birth location | Home  Hospital  ? | 1348 (29.6%)  3137 (68.9%)  68 (1.5%) | 384 (23.9%) 1195 (74.5%) 25 (1.6%) | 0.001^*^ |
| **Family** | |  |  |  |
| Years of education mother | Mean (SD) | 9.5 (3.8) | 7.7 (4.0) | <0.001^*^ |
| Ethnic origin | Dutch | 2968 (65.2%) | 697 (43.5%) | <0.001^*^ |
|  | Non Dutch | 1583 (34.8%) | 903 (56.3%) |  |
|  | Turkish | 156 (3.4%) | 115 (7.2%) |  |
|  | Moroccan | 271 (6.0%) | 200 (12.5%) |  |
|  | African  Other: Western  Other: Non-Western | 234 (5.1%)  549 (12.1%)  373 (8.2%) | 167 (10.4%)  191 (11.9%)  230 (14.4%) |  |

*significant
